# Intramural enterolith mimicking malignancy in the terminal ileum: a rare case report

**DOI:** 10.1093/jscr/rjag449

**Published:** 2026-06-27

**Authors:** Jaber Ali Al-Faifi, Ghaida Hassan Alharbi, Ghada Ibraheem Alshaher, Musab Alamri, Rahaf Ibrahim Alshaher

**Affiliations:** Department of General and Colorectal Surgery, King Saud Medical City, Imam Abdulaziz bin Mohammed bin Saud Road, Al Shumaisi District, Riyadh 12746, Ar Riyad Province, Saudi Arabia; Department of General Surgery, King Saud Medical City, Imam Abdulaziz bin Mohammed bin Saud Road, Al Shumaisi District, Riyadh 12746, Ar Riyad Province, Saudi Arabia; Department of General Surgery, King Saud Medical City, Imam Abdulaziz bin Mohammed bin Saud Road, Al Shumaisi District, Riyadh 12746, Ar Riyad Province, Saudi Arabia; Department of Radiology, King Saud Medical City, Imam Abdulaziz bin Mohammed bin Saud Road, Al Shumaisi District, Riyadh 12746, Ar Riyad Province, Saudi Arabia; College of Medicine, King Khalid University, King Abdullah Road, Al Qariqer District, Abha 62529, Asir Province, Saudi Arabia

**Keywords:** enterolithiasis, intramural enterolith, terminal ileum, small bowel mass, intestinal perforation

## Abstract

Enterolithiasis is an uncommon condition characterized by the formation of calculi within the intestinal lumen or bowel wall, often associated with intestinal stasis or structural abnormalities. Although many enteroliths remain asymptomatic, impacted stones may cause obstruction, inflammation, or perforation and can mimic malignancy or chronic infection. We report a case of a 54-year-old male presenting with constitutional symptoms and imaging findings suggestive of a terminal ileal mass with contained perforation. Further evaluation demonstrated an intramural enterolith. Because malignancy could not be excluded, surgical resection was performed. Histopathology confirmed inflammatory changes surrounding an impacted enterolith without malignancy. This case highlights the diagnostic challenge of enterolithiasis when presenting as a mass-like lesion and emphasizes the importance of considering benign etiologies in complex abdominal presentations.

## Introduction

Enterolithiasis refers to the formation of stone-like concretions within the gastrointestinal tract and is considered a rare and under-recognized condition [1]. Enteroliths may form primarily within the bowel due to stasis, altered motility, diverticula, or strictures, or secondarily from migration of calculi originating outside the intestine, such as in gallstone ileus [2]. Their composition varies depending on luminal pH and intestinal environment, with bile acid–based stones more commonly forming in proximal segments and calcium-based stones forming in distal small bowel regions [3]. Most enteroliths pass spontaneously without clinical consequence; however, larger stones may become impacted and lead to intestinal obstruction, hemorrhage, or perforation [4]. The clinical presentation is often nonspecific and may mimic inflammatory, infectious, or neoplastic processes [2]. Radiologic imaging, particularly contrast-enhanced computed tomography (CT), plays a crucial role in detection and evaluation of associated complications, although definitive diagnosis may still be challenging [2]. Surgical intervention becomes necessary when obstruction persists, perforation occurs, or malignancy cannot be excluded [4].

## Case presentation

A 54-year-old male with a known history of hypertension and a long smoking history presented with acute-on-chronic right lower quadrant abdominal pain. The pain was progressive and associated with constipation for 3 days. He also reported systemic symptoms, including intermittent fever, unintentional weight loss exceeding 5 kg over one month, anorexia, and night sweats. He had recent exposure to livestock and unpasteurized dairy products.

On examination, the patient was hemodynamically stable. Abdominal examination revealed a firm, fixed, tender mass in the right lower quadrant measuring ~7 × 7 cm. There were no signs of generalized peritonitis. Digital rectal examination was unremarkable.

Laboratory investigations demonstrated leukocytosis with neutrophilia, normocytic anemia, and thrombocytosis. Renal function was impaired, and liver enzymes were mildly elevated. Carcinoembryonic antigen was slightly elevated. Blood cultures were positive for Brucella species, while other infectious workup, including tuberculosis testing, was negative.

Review of prior imaging from two years earlier revealed mild terminal ileal wall thickening with a large fecalith but no obstruction. Repeat contrast-enhanced CT demonstrated significant disease progression, including extensive terminal ileal wall thickening, a large soft tissue mass, and a localized collection consistent with contained perforation. These findings raised differential considerations, including lymphoma, intestinal tuberculosis, or an impacted fecalith with secondary perforation (Fig. 1).

**Figure 1:**
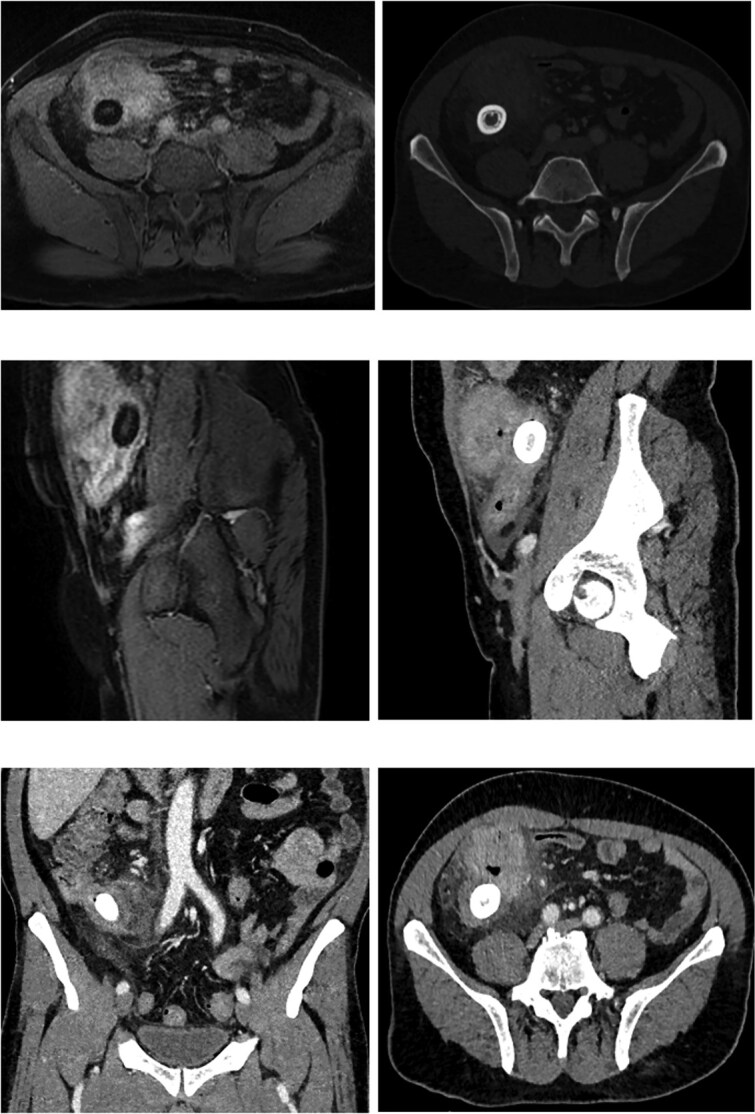
CT and MRI showing progressive terminal ileal wall thickening with mass and contained perforation; MRI demonstrates intraluminal enterolith.

Colonoscopy was attempted but could not traverse the ileocecal region due to severe stenosis, with an obstructing lesion highly suspicious for malignancy (Fig. 2). Magnetic resonance imaging (MRI) of the abdomen and pelvis demonstrated a terminal ileal mass containing a well-defined intraluminal structure consistent with an enterolith, surrounded by inflammatory changes.

**Figure 2:**
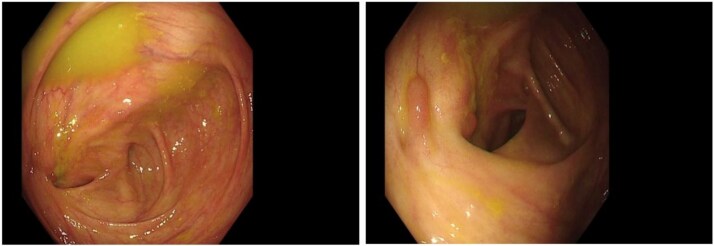
Colonoscopy showing severe ileocecal stenosis with non-passable scope and suspicious obstructing lesion.

Given persistent symptoms, progressive imaging findings, and inability to exclude malignancy, the patient underwent laparoscopic right hemicolectomy. Intraoperatively, a large inflammatory mass involving the terminal ileum with dense adhesions to adjacent structures was identified (Fig. 3). No distant metastases or peritoneal deposits were observed.

**Figure 3:**
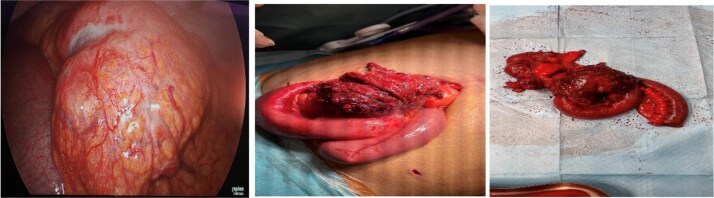
Intraoperative view showing large terminal ileal mass with dense inflammatory adhesions.

Histopathological examination demonstrated transmural inflammation, microabscess formation, fistulous tracts, and foreign body giant cell reaction surrounding an impacted enterolith. No dysplasia or malignancy was identified. The postoperative course was uneventful, and the patient recovered without complications.

## Discussion

Enteroliths vary widely in size, number, and composition and are broadly classified as primary or secondary [1]. Primary enteroliths form within the intestine, usually in areas of luminal stasis such as diverticula or strictures [5]. Secondary enteroliths develop outside the bowel and migrate through fistulous connections, most commonly in gallstone ileus [6]. Clinical manifestations depend on stone size and location. Small enteroliths are frequently asymptomatic, while larger stones may cause intermittent abdominal pain, partial obstruction, or acute bowel obstruction [4]. In rare cases, chronic inflammation surrounding an impacted stone may produce a mass-like lesion with surrounding fibrosis and localized perforation, closely mimicking malignancy [2]. Radiologic diagnosis can be difficult because many enteroliths are radiolucent and not easily detected on plain radiographs [2]. Cross-sectional imaging is therefore essential for identifying associated inflammatory changes, obstruction, or perforation [2]. Even with advanced imaging, differentiation from malignancy or infection may remain uncertain, particularly when systemic symptoms or elevated tumor markers are present. Management depends on clinical presentation and severity of complications. Conservative measures may be considered in partial obstruction; however, persistent obstruction, perforation, or diagnostic uncertainty requires surgical intervention [4]. Surgical options include enterotomy with stone extraction or segmental bowel resection when extensive inflammation or suspected malignancy is present [4]. Histopathological evaluation remains essential to exclude neoplastic disease. In the present case, constitutional symptoms, positive blood cultures, progressive mass formation, and contained perforation created a complex diagnostic picture strongly suggestive of malignancy or chronic infection. The presence of an intramural enterolith with extensive inflammatory reaction ultimately explained the findings. Surgical resection provided both definitive treatment and diagnosis.

## Conclusion

Enterolithiasis is an uncommon but clinically significant condition that may present with nonspecific symptoms and radiologic findings mimicking malignancy. Intramural enteroliths can produce mass-like lesions, bowel obstruction, and contained perforation, creating substantial diagnostic uncertainty. This case emphasizes the importance of maintaining a broad differential diagnosis when evaluating atypical abdominal masses and highlights the role of surgical intervention when malignancy cannot be excluded. Early recognition and appropriate management are essential to prevent complications and ensure accurate diagnosis.
